# *Syzygium aromaticum* (clove buds) as a natural antibacterial agent: a promising alternative to combat multidrug-resistant bacteria

**DOI:** 10.3389/fmicb.2025.1674590

**Published:** 2025-10-14

**Authors:** Hira Tariq, Abdulrahman Mohammed Alhudhaibi, Emad M. Abdallah

**Affiliations:** ^1^Institute of Microbiology and Molecular Genetics, University of Punjab, Lahore, Pakistan; ^2^Department of Biology, College of Science, Imam Mohammad Ibn Saud Islamic University (IMSIU), Riyadh, Saudi Arabia; ^3^Department of Biology, College of Science, Qassim University, Buraydah, Saudi Arabia

**Keywords:** antibacterial activity, *Syzygium aromaticum*, multidrug-resistant pathogens, medicinal plants, natural drugs, health

## Abstract

Antibiotic resistance is a critical threat to modern medicine, necessitating new strategies against multi-drug resistant bacteria (MDR). This narrative review evaluates the published evidence on *Syzygium aromaticum* (clove) and its principal phytochemicals, with particular focus on activity against MDR pathogens. We describe the chemical profile, notably eugenol, quercetin, kaempferol, *β*-caryophyllene, and *α*-humulene, and summarize reported *in vitro* activity and substantial biofilm inhibition (up to about 90%) against clinically relevant MDR bacteria such as *Escherichia coli*, *Staphylococcus aureus*, and *Klebsiella pneumoniae*. Mechanistically, clove phytochemicals act through a hierarchical cascade in which membrane perturbation is the primary initiating event. Lipophilic constituents (notably eugenol) partition into and disorder the phospholipid bilayer, causing rapid loss of membrane potential, K^+^/ATP efflux and collapse of the proton-motile force (PMF). This primary membrane failure then enables secondary intracellular consequences, impaired electron transport and tricarboxylic acid cycle (TCA-cycle) enzyme activity, increased electron leakage with reactive oxygen species (ROS) generation, macromolecular damage (lipid peroxidation, protein oxidation, DNA strand injury), and functional inhibition of energy-dependent efflux, which together produce bactericidal outcomes and potentiate synergy with conventional antibiotics. Importantly, phytochemicals from clove frequently act synergistically with conventional antibiotics, lowering antibiotic MICs by approximately 4–128-fold and enhancing agents including colistin, imipenem, and amikacin. We evaluate formulation approaches (nano-emulsions, liposomes, solid-lipid nanoparticles) aimed at improving delivery and bioavailability, and we review limited preclinical and early clinical observations that suggest benefits in settings such as ventilator-associated pneumonia and MRSA wound healing. Critical translational gaps remain. Robust *in vivo* efficacy data, standardized pharmacokinetic and toxicology characterization, stability studies, and rigorous clinical trials are urgently needed. We conclude by proposing a focused research roadmap to validate and responsibly translate clove-derived candidates as adjuncts to existing antibiotic regimens.

## 1 Introduction

Antimicrobial resistance has emerged as a significant global health concern in the 21st century. Projections indicate a substantial increase in mortality due to drug-Resistant pathogens, with approximately 1.91 million deaths directly attributable to it and 8.22 million associated deaths anticipated by 2050 (Naghavi et al., 2024). The World Health Organization (WHO) has classified antimicrobial resistance as a leading global public health threat (Chawla et al., 2022). The overuse and misuse of antibiotics in humans and animals create selection pressure, leading to the emergence and persistence of resistant bacterial strains (Elhaddadi et al., 2024). Gram-negative bacterial pathogens are responsible for the majority of hard-to-treat illnesses, and they are resistant to almost all antimicrobials (Darwish et al., 2022). The CDC (Centers for Disease Control and Prevention) estimates that antibiotic-resistant infections in the United States affect over 2.8 million individuals annually, resulting in approximately 35,000 deaths (Sulong et al., 2024).

Antimicrobial resistance (AMR) can be defined as the capacity of microorganisms, such as bacteria, fungi, parasites, and viruses to survive exposure to antimicrobial agents such as antibiotics, antifungals, antivirals, and antiparasitic, thereby reducing or nullifying treatment efficacy (Tang et al., 2023). While multi-drug resistant (MDR) bacteria are a subset of AMR; which refers to bacteria that display non-susceptibility to at least one antibiotic in three or more different classes of antibacterial drugs (Alkofide et al., 2020). The increasing burden of AMR and MDR pathogens not only leads to heightened healthcare costs and reduced productivity but is also projected to have severe economic implications, potentially raising global costs to $100 trillion annually by 2050 (Domínguez et al., 2021). MDR bacteria employ various resistance mechanisms, including the production of antibiotic-degrading enzymes and extracellular structures such as biofilms, which inhibit antibiotic penetration and action (Muhammad and Shoge, 2023). Horizontal gene transfer further exacerbates the problem by facilitating the spread of resistance genes, making treatment options increasingly limited (Rao et al., 2023). Horizontal gene transfer, via conjugation, transformation and phage-mediated transduction, rapidly mobilizes resistance determinants across strains, species and ecological niches and creates a shared resistome that accelerates the emergence of MDR pathogens (Lawani et al., 2023). Therefore, multi-target agents or multi-component therapies, which require simultaneous compromise of independent cellular pathways, are a genetically justified alternative to single-target antibiotics.

**Figure 1:**
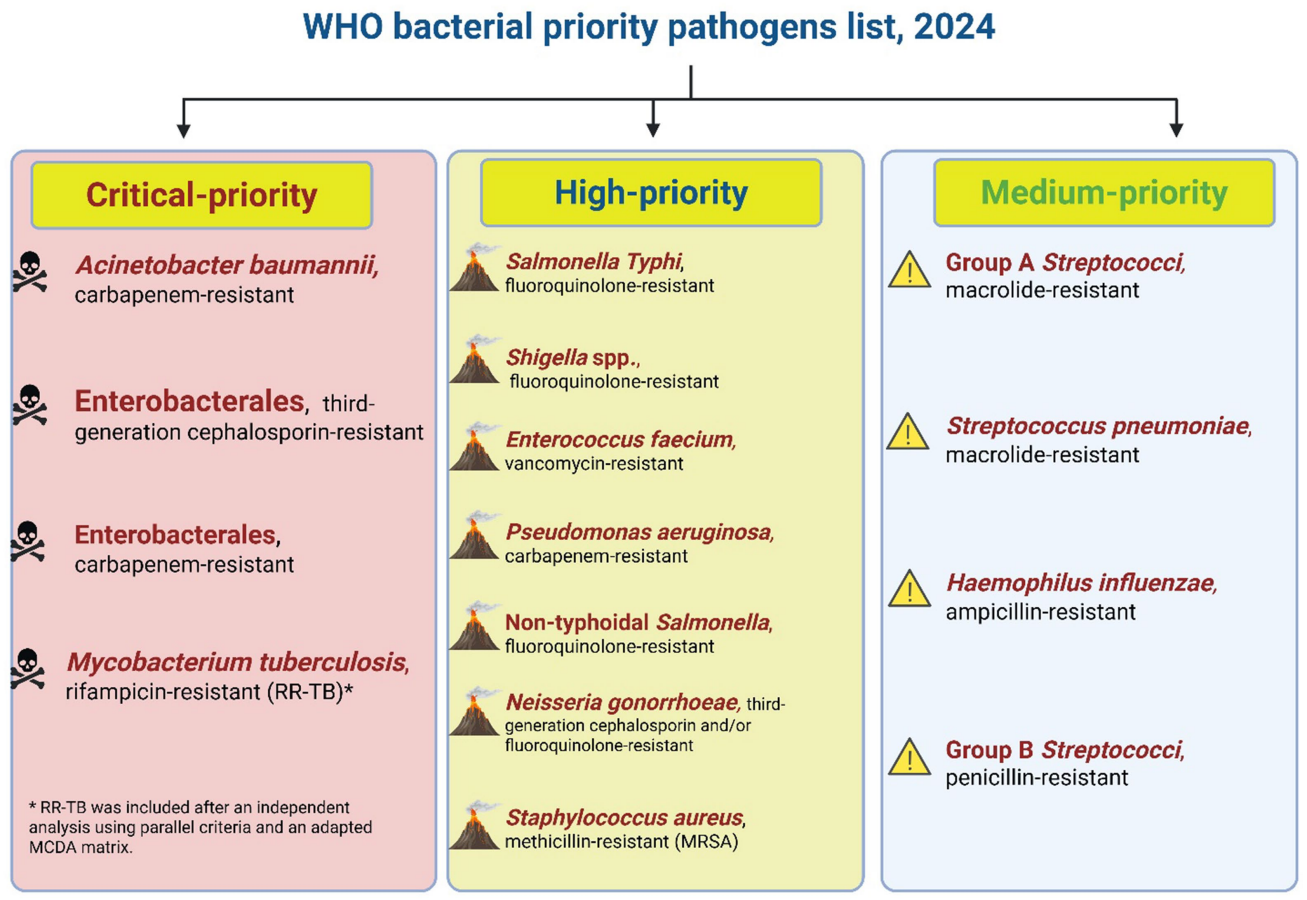
Priority levels for antibiotic-resistant bacteria according to the 2024 WHO report. This figure synthesizes the WHO’s 2024 prioritization of bacterial pathogens into three urgency tiers and is intended as a decision-making framework: it highlights where the greatest unmet clinical need and research investment are required to reduce morbidity and mortality from antimicrobial-resistant infections. Data source: World Health Organization (2024). (This figure was created by the author using BioRender).

To address this crisis, the World Health Organization (WHO) has emphasized the ineffectiveness of current antibiotics and underscored the urgent need to develop alternative strategies to combat common pathogens that now threaten medical advancements (Gupta and Bhandary, 2024). The global shortage of novel antibiotics represents a significant research gap that threatens modern medicine in the face of escalating antibacterial resistance and the antibiotic development pipeline remains alarmingly sparse. This crisis demands unified global political action. UN member states must prioritize AMR and MDR nationally while mobilizing sustainable funding and bridging innovation with equitable access. In 2024, the World Health Organization (WHO) released its updated Bacterial Priority Pathogens List (BPPL), identifying 15 families of life-threatening, antibiotic-resistant bacteria stratified into critical, high, and medium priority categories (Figure 1). This list serves as an urgent global alert, underscoring the escalating threat these pathogens pose to human health and healthcare systems. It highlights the pressing need for both the discovery of novel antibacterial agents and innovative strategies, such as synergistic combinations to restore the efficacy of existing antibiotics, to curb the spread of these formidable drug-resistant infections (Jesudason, 2024). Without coordinated investment and policy alignment, scientific breakthroughs will fail to reach those most vulnerable (Balasegaram et al., 2024).

Medicinal plants have served as the primary source of therapeutic agents for treating various ailments, including pathogenic infections, since ancient times. Their use and development as anti-infective agents persisted throughout human civilizations. However, with the advent of the modern era, a paradigm shift occurred as scientists increasingly relied on synthetic and semi-synthetic antibiotics for combating microbial infections (Abdallah, 2011). Since the 1960s, antibiotic resistance phenomenon has progressively increased, while the emergence of new antibiotic classes has markedly diminished. This increasing worry poses a renewed danger of epidemics and bacterial diseases, potentially resulting in significant, enduring consequences for human history (Abdallah et al., 2023). A substantial amount of literature has recorded the antibacterial potential of several medicinal plants. A total of 6,083 publications published from 1946 to 2019 examined the antibacterial efficacy of various plant species. The most frequently reported plant families were Lamiaceae, Asteraceae, and Fabaceae, with *Cinnamomum verum*, *Thymus vulgaris*, and *Rosmarinus officinalis* identified as the most extensively studied species (Chassagne et al., 2021). Among different plat extracts, the plant essential oils (EOs) are widely recognized for their biologically active chemical constituents, which exhibit notable therapeutic properties, underscoring their potential as promising candidates in antibacterial agent’s development. Accordingly, considerable scientific attention has been directed toward investigating their antimicrobial efficacy, with studies emphasizing their capacity to serve as sources of novel therapeutic agents for combating infectious pathogens. This growing body of research underscores their viability as alternatives or adjuncts to conventional antimicrobial treatments (Al-Mijalli et al., 2025).

**Figure 2:**
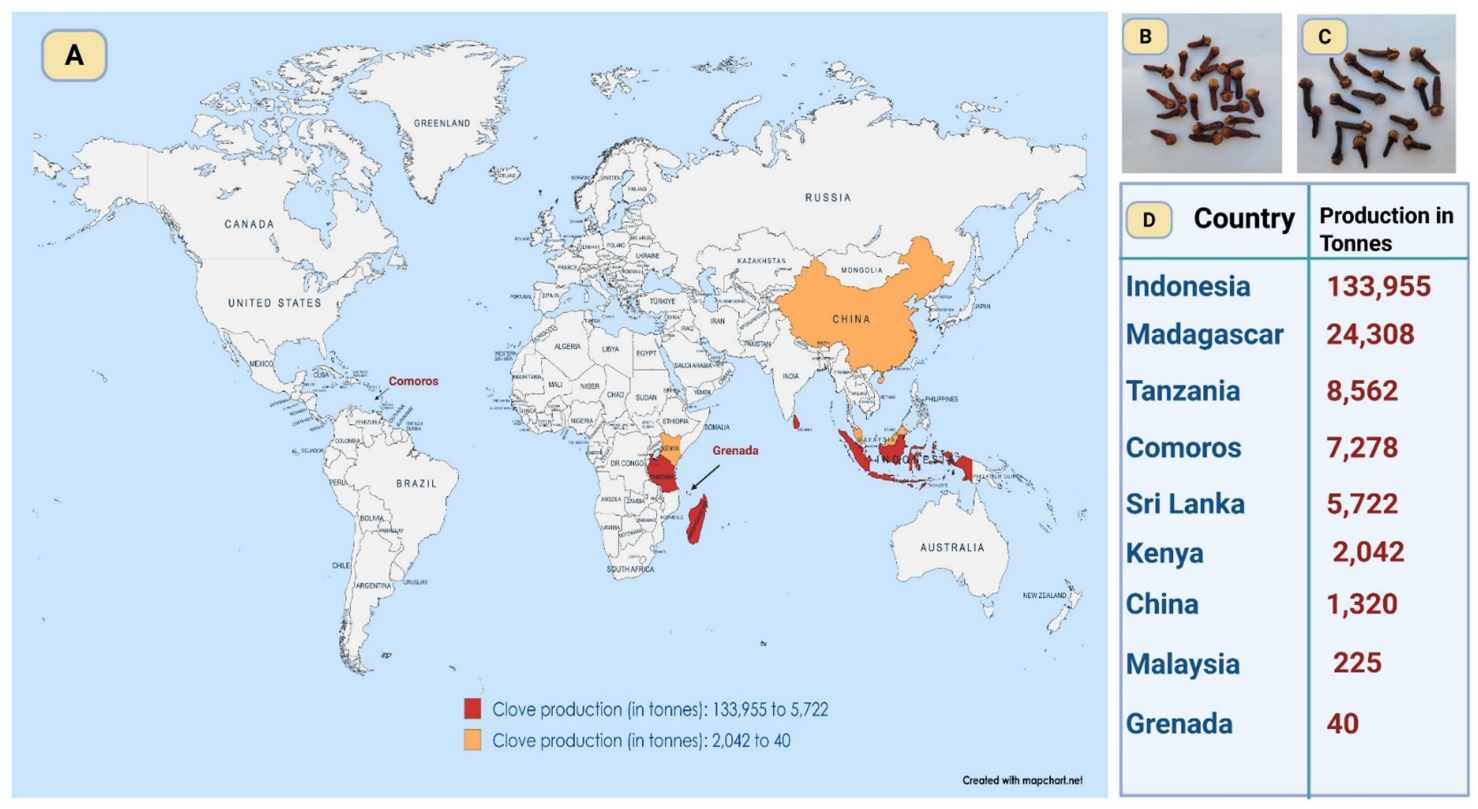
Global clove cultivation at a glance. This map depicts major clove-producing countries **(A)**, linking production hotspots with regional conditions that shape phytochemistry, antimicrobial efficacy, and sourcing potential. It further contrasts Sri Lankan cloves, lighter and milder **(B)**, with Indonesian cloves, darker and more pungent **(C)**, and presents the FAO’s latest (2022) production estimates in tonnes **(D)**. (Source of data: https://www.fao.org/faostat/en/#data/QCL).

*Syzygium aromaticum* (L.) Merr. and L. M. Perry, is a significant medicinal plant used in Asia for therapeutic and culinary applications. The blossoming buds are known as cloves, which are dried and widely traded. Its usage dates back to approximately 2000 years, with historical records indicating its employment by the Chinese and Indians for the treatment of numerous illnesses, including respiratory and stomach ailments (Bhowmik et al., 2012). Cloves also cultivated in many countries in tropical regions including Indonesia, Madagascar, Tanzania, Comoros, Sri Lanka, Comoros, Kenya, China, Malaysia, and Grenada (Figure 2). Cloves, originating from Maluku Island in Indonesia, contains eugenol as its primary active ingredient, which is responsible for both its aroma and therapeutic properties (Kamatou et al., 2012). In Ayurvedic medicine, clove has long been used to alleviate pain and inflammation, as well as to combat various microbial infections (Mugnaini et al., 2020). Its traditional applications extend to the treatment of oral and dental infections and periodontal disease, a noteworthy area of exploration (Kaur and Chandrul, 2017). Given that clove oil has emerged as a potential treatment for common oral infections, as supported by Marya *et al* (Marya et al., 2012), who demonstrated its inhibitory effect on tooth decalcification, its broader therapeutic applications warrant further investigation. Moreover, studies on clove oil suggest that it effectively addresses pain, inflammation, and microbial infections, in line with its traditional medicinal uses. It has also been employed as an analgesic, for treating respiratory tract infections, and to enhance digestion. The antimicrobial properties of clove have garnered increasing attention in recent years, driven by the escalating global problem of antibiotic resistance. Previous research has demonstrated that clove oil is effective against both Gram-positive and Gram-negative bacteria, including *Escherichia coli* and *Staphylococcus* spp., and various MDR traits were initially identified in isolates from food animals and later transitioned into clinical environments (Lv et al., 2024). Additionally, clove oil has shown efficacy against the notorious nosocomial pathogen *Pseudomonas aeruginosa,* which severely affects immunocompromised patients (Costa et al., 2022). The mechanism of action of clove oil is believed to involve disruption of bacterial cell membranes, biofilm breakdown, and interference with metabolic pathways (Bhattacharjee et al., 2023). Combining clove oil with antibiotics has been shown to enhance the efficacy of the latter through synergistic effects (Attaallah Ibrahim and Kadhim Mohammed, 2023). Moreover, cloves phytochemicals exhibit anti-inflammatory and antioxidant properties, with their phytochemical components, such as flavonoids and phenolics, further enhancing their therapeutic potential (AlKhafaji et al., 2024).

The aim of this review is to critically explore the antibacterial potential of extracts of clove buds of *Syzygium aromaticum*, particularly its essential oilsas a natural alternative to combat MDR bacteria. Given the escalating global health crisis posed by MDR pathogens and the limitations of conventional antibiotics, additionally, it highlights the synergistic effects of clove extracts with existing antibiotics, offering insights into its potential role in mitigating antimicrobial resistance and advancing innovative treatment strategies.

## 2 Phytochemical composition of clove essential oil

**Figure 3:**
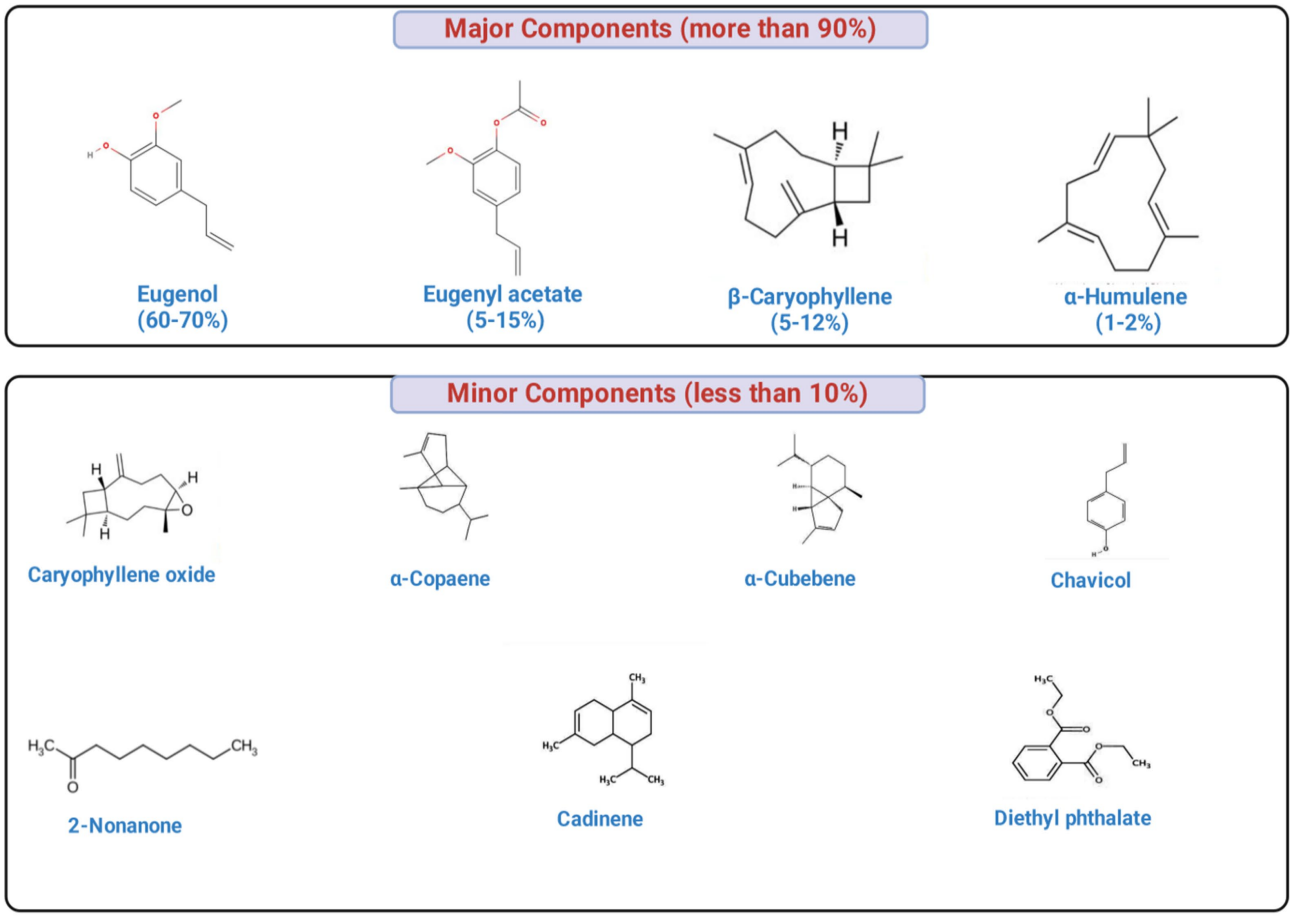
Major and minor bioactive phytochemicals in clove essential oil. The oil is dominated by eugenol (70–95%), with eugenyl acetate (up to 20%), β-caryophyllene (12–17%), and *α*-humulene (1–2%) as additional key constituents, while minor components (<10%) include caryophyllene oxide, α-copaene, α-cubebene, chavicol, cadinene, 2-nonanone, diethyl phthalate, and related compounds. Notably, eugenol and several terpenoids exhibit strong antibacterial activity through membrane disruption, leakage of intracellular contents, and inhibition of essential cellular processes.

Clove essential oil is characterized by a complex phytochemical profile (up to 30 compounds were identified in cloves), with eugenol (70–95%) as the predominant constituent, followed by eugenol acetate (≤20%) and *β*-caryophyllene (12–17%), collectively constituting 15–20% of the total oil content (Strižincová et al., 2022). Other minor or trace components comprise less than 10% of the composition, including caryophyllene oxide, diethyl phthalate, cadinene, 4-(2-propenyl)-phenol, *α*-copaene, α-cubebene and chavicol, among others (Haro-González et al., 2021). The concentrations of the phytochemical constituents of the clove greatly vary based on geographic origin, extraction technique, and environmental conditions. Figure 3 showing the major bioactive components of clove.

### 2.1 Eugenol

Eugenol (4-allyl-2-methoxyphenol), an aromatic phenolic compound, is the principal bioactive constituent of clove, with leaf extracts yielding up to 9381.7 mg per 100 g (Nejad et al., 2017). This compound is distinguished by its viscous yellow appearance, pronounced aroma, and lipophilic properties, which facilitate interactions with biological membranes (Ulanowska and Olas, 2021; Zhai et al., 2022). The hydroxyl group in its molecular structure confers antimicrobial activity via disruption of bacterial membrane integrity and inhibition of protease function (Pilong et al., 2023). Additionally, eugenol exhibits potent antioxidant properties, scavenging reactive oxygen species (ROS) and upregulating endogenous antioxidant enzymes (e.g., superoxide dismutase, catalase), thereby mitigating oxidative stress (Ma et al., 2021). Eugenol demonstrates antibacterial activity against *Shigella flexneri* by inducing oxidative damage. It suppresses superoxide dismutase activity, raising intracellular ROS levels. This oxidizes the cell membrane, compromising its barrier function and causing ATP leakage and depolarization (Khwaza and Aderibigbe, 2025).

In antibacterial applications, eugenol (3 mM) significantly reduces bacterial metabolic activity and biofilm formation, attributed to interference with quorum-sensing pathways (Olszewska et al., 2020). Its anti-inflammatory and analgesic properties underpin its therapeutic use in dentistry for post-procedural care (e.g., root canal therapy, tooth extraction) (Veerapandi et al., 2022). Mechanistic studies in rheumatoid arthritis models demonstrate eugenol-induced apoptosis of fibroblast-like synoviocytes, suppression of pro-inflammatory cytokines (e.g., IL-6, TNF-*α*), and inhibition of cell migration (Ahmad et al., 2021; Damasceno et al., 2024). Eugenol’s anticancer potential is evidenced by its capacity to induce apoptosis and cell cycle arrest across multiple cancer lineages. In colorectal carcinoma, eugenol modulates Wnt/*β*-catenin signaling, while in cervical cancer, it synergizes with cisplatin to enhance chemosensitivity through downregulation of anti-apoptotic proteins (Bcl-2) (Fathy et al., 2019; Zari et al., 2021; Islam et al., 2022). Clinically, eugenol is utilized as a topical antiseptic and anesthetic agent (Dable-Tupas et al., 2023).

Eugenol should not be viewed merely as a constituent of clove, but rather as a prototypical phytophenolic derivative that exemplifies the multimodal antimicrobial potential of polyphenols. As demonstrated by Bayode et al. (2024), phytophenols exert broad-spectrum activity through multiple mechanisms, including disruption of microbial membranes, inhibition of efflux pumps, interference with essential enzymes such as DNA gyrase, and modulation of stress responses via antioxidant effects. This multifaceted mode of action confers intrinsic, multi-target efficacy against MDR pathogens. Conceptualizing eugenol within this framework underscores its translational relevance and supports the rationale for developing multi-component phytochemical strategies to combat clinical “superbugs.”

### 2.2 Eugenol acetate

Eugenol acetate, a phenylpropanoid derivative, constitutes ≤20% of CEO and demonstrates enhanced bioactivity relative to eugenol in prostate and oral squamous carcinoma models (Das et al., 2019). Its antioxidant capacity (90.31% DPPH scavenging at 35 μg/mL) and anti-inflammatory effects (inhibition of TNF-*α*, IL-1*β*) are well-documented (Adaramola and Onigbinde, 2016; El Ghallab et al., 2020). Ultrasound-assisted hydrotropic extraction (158 W, 38°C, 30 min) yields 20.04% eugenol acetate, outperforming Soxhlet methods (5.72%) by reducing solvent use and extraction time (Ali et al., 2021; Ahmed et al., 2022). Eugenyl acetate exhibits toxicity against brine shrimp at 0.3 μg/mL and potent larvicidal effects against the yellow fever mosquito (*Aedes aegypti*) (LC₅₀: 0.1 mg/mL) by targeting octopaminergic receptors. Its diverse properties, including antioxidant, antimicrobial, antitumor, and insecticidal activities, make it valuable for applications in food preservation (Haro-González et al., 2021).

### 2.3 *β*-Caryophyllene

β-Caryophyllene (about 13% of cloves essential oils), a bicyclic sesquiterpene, exhibits broad-spectrum antimicrobial activity and anticancer effects via apoptosis induction in ovarian and lung cancers (Santolin et al., 2021; Tarhan, 2021). In neurodegenerative models, it activates NQO1protien, ameliorating oxidative damage in Parkinson’s disease (Nisar et al., 2021). β-Caryophyllene from clove extract shows potent antimicrobial activity against *Helicobacter pylori*, significantly reducing bacterial load both *in vitro* and *in vivo*. Beyond its direct bactericidal effects, it also inhibits gastric mucosal inflammation, highlighting its potential as a promising anti- *Helicobacter pylori* agent (Jung et al., 2020). Methods of extractions have impact on the quantity extracted from the cloves, hydrodistillation maximizes β-caryophyllene yield (36.94%),

### 2.4 *α*-humulene

α-Humulene is a sesquiterpene that constitutes approximately 2.75% of clove buds, although its concentration may vary slightly across different clove varieties. The characteristic woody aroma of clove is largely attributed to this compound (Amelia et al., 2017). Despite being a less abundant constituent, *α*-humulene demonstrates notable anti-inflammatory and antiproliferative activities in colon, prostate, and breast cancers through inhibition of the NF-κB pathway (Ambrož et al., 2019). In addition, essential oils enriched with α-humulene display antibacterial activity, supporting its therapeutic relevance. Safety assessments reveal that α-humulene is non-toxic to fibroblast and macrophage cell lines at concentrations up to 400 μg/mL, while higher doses induce cytotoxic effects (Jang et al., 2020). Beyond these properties, α-humulene has also been reported to exhibit antifungal, gastroprotective, antiallergic, and antiparasitic activities (Dalavaye et al., 2024).

### 2.5 Other minor components

Other minor constituents of clove buds essential oil comprise various compounds, each constituting less than 10% of the total composition. These include caryophyllene oxide, α-copaene, α-cubebene, chavicol, diethyl phthalate, 2-nonanone, and several others (Haro-González et al., 2021; Ahamad, 2023; Figure 3). Clove essential oil contains several minor constituents, including 0.91% α-terpineol, 0.40% D-limonene, 0.26% p-cymene, 0.21% *γ*-muurolene, 0.13% γ-terpinene, 0.12% geranyl acetate, 0.11% 2-nonanone, 0.11% linalool, 0.10% terpinolene, and 0.10% germacrene B (Ahamad, 2023). Therefore, the minor constituents of clove essential oil primarily belong to four chemical classes: monoterpenes (e.g., D-limonene, α-terpineol, linalool), sesquiterpenes (e.g., γ-muurolene, germacrene B), phenylpropanoids (e.g., p-cymene, chavicol), and ketones/esters (e.g., 2-nonanone, geranyl acetate). These compounds contribute to the oil’s aroma and bioactivity.

## 3 Antibacterial mechanisms and synergy of cloves

### 3.1 Inhibition of bacterial growth and biofilm disruption

Natural plants containing essential oils have antimicrobial properties and serve as a potential source for addressing antibiotic resistance (Cui et al., 2020). Clove essential oil is a potent antimicrobial agent, primarily due to its major chemical constituent, eugenol. Other components present in lower quantities, such as caryophyllene, eugenol acetate, and benzyl salicylate, may contribute synergistically to its antimicrobial activity (Pateiro et al., 2021).

A previous study evaluated the antibacterial effects of clove essential oil against Gram-negative pathogens, including *E. coli*, *Klebsiella* sp., *Enterobacter* sp., *Citrobacter* sp., *Proteus* sp., *Pseudomonas* sp., and *Acinetobacter* sp. The inhibition zones at a concentration of 100 mg/mL were reported as 17, 16, 17, 18, 19, 14, and 18 mm, respectively. All tested strains were screened for *β*-lactamase production (ESBL, MBL, and AmpC enzyme), with *Proteus* species exhibiting the highest susceptibility. These findings suggest that clove essential oil has varying antibacterial potential against uropathogens and could be explored for the development of novel antimicrobial agents to address the growing issue of antibiotic resistance (Faujdar et al., 2020).

**Figure 4:**
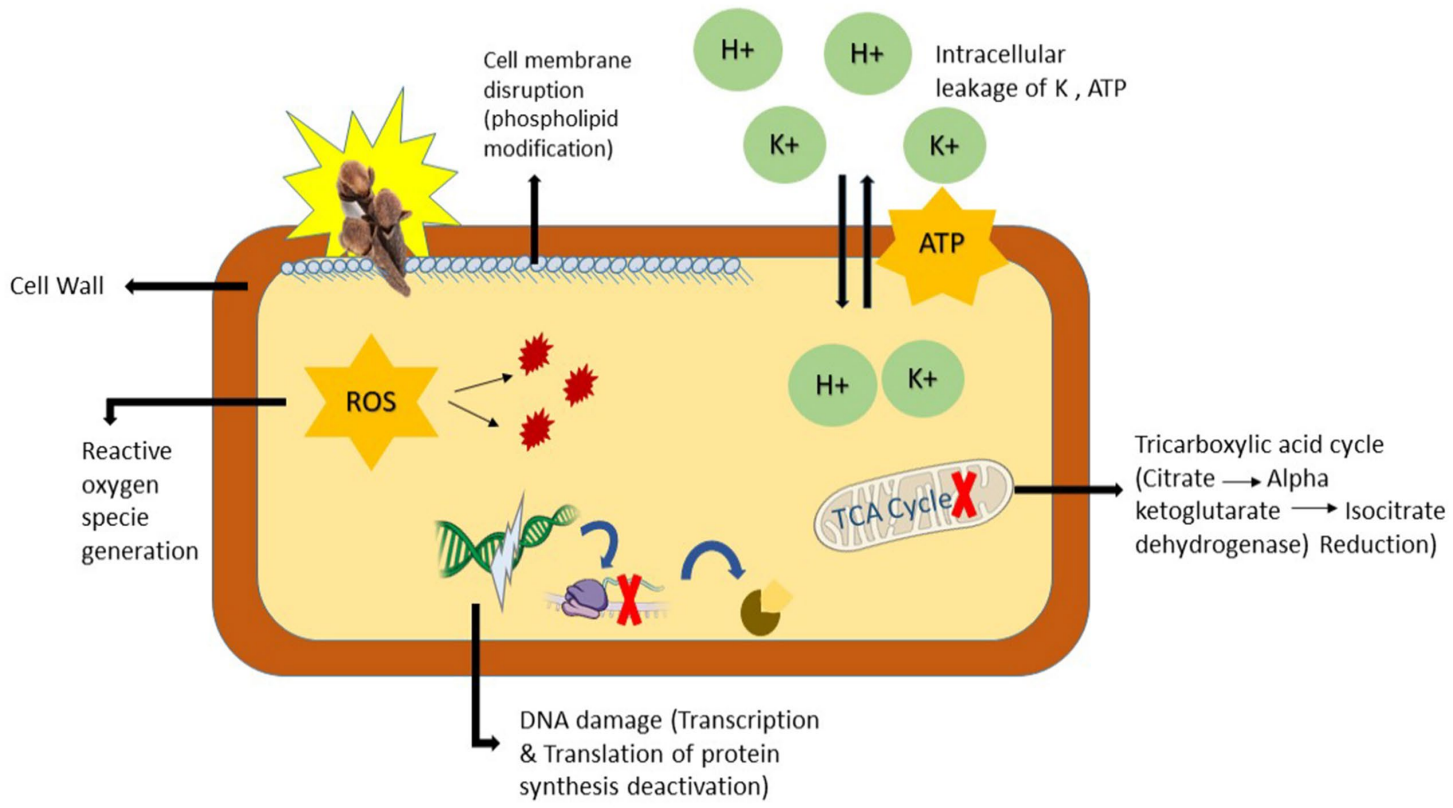
Proposed antibacterial mechanisms of clove essential oil. This figure is a schematic representation of the cascade model of antimicrobial action of clove phytochemical compounds, highlighting eugenol-mediated membrane disruption, ion and ATP leakage, inhibition of TCA-cycle enzymes, ROS overproduction, and consequent macromolecular damage leading to cell death. (This figure was created by the author using power point).

Most natural antibacterial agents exert their activity through the breakdown of the cell wall and membrane (Hu et al., 2019), inhibition of biofilm formation (Latifah-Munirah et al., 2015), and interference with DNA replication processes (Rajkowska et al., 2017). The antibacterial properties of clove essential oils are mainly attributed to eugenol, the major constituent of clove buds. Eugenol has shown strong bactericidal activity against MDR *Streptococcus suis*. At a 15% oil concentration, the maximum zones of inhibition were observed, and time-kill analysis revealed that *S. suis* was completely eradicated within 15 min. Scanning electron microscopy (SEM) confirmed atypical cellular morphology and cell membrane lysis (Wongsawan et al., 2019). The chemical composition of clove essential oil contains (–OH) groups at meta and ortho positions, which interact with the cytoplasmic membrane, disrupt phospholipids, and subsequently inhibit protein translocation, phosphorylation, electron transport, and other enzymatic activities, ultimately leading to cell death (Shahbazi, 2019; Figure 4).

The *in vitro* minimum inhibitory concentration (MIC) and minimum bactericidal concentration (MBC) assays indicate that cloves essential oils are more effective against *Listeria innocua* and *S. aureus* than against Gram-negative bacteria. SEM micrographs of *E. coli* and *L. innocua* eradicated with cloves essential oils at MIC values of 6.25 mg/mL and 1.56 mg/mL, respectively, for 12 h revealed increased membrane permeability, disrupted integrity, cytoplasmic leakage, and cell lysis (Behbahani et al., 2019).

Gram-positive bacteria are more susceptible to essential oils due to their single-layered peptidoglycan cell wall, which offers weak resistance to antibacterial agents (Zhao et al., 2021). In contrast, Gram-negative bacteria possess an outer membrane composed of lipopolysaccharides, peptidoglycan, and various proteins, forming a complex structure that hinders the diffusion of lipophilic compounds (Kim et al., 2019). Beyond simple structural differences, a physicochemical explanation explains differential susceptibility: the lipopolysaccharide (LPS)-rich outer membrane of Gram-negative bacteria forms a hydrated, highly anionic and amphipathic barrier that actively limits partitioning of hydrophobic phytochemicals into the inner phospholipid bilayer. As Lawani et al. (2023) emphasize, this LPS layer does not merely act as a passive wall but repels or reduces the local concentration of lipophilic molecules such as eugenol at the membrane surface, thereby diminishing their ability to insert into and disorder bacterial membranes. This physicochemical repulsion helps explain why covle essential oils often shows lower *in vitro* potency against Gram-negative strains unless delivery (e.g., nano-emulsions) or permeabilizing co-agents are used. Moreover, one study also demonstrated the antibacterial mechanism of eugenol, showing that it alters membrane permeability nonspecifically, interfering with ion and ATP transport. This was evidenced by a significant release of K^+^ ions in phosphate-buffered saline (PBS) in *Listeria monocytogenes*, increasing membrane permeability (Marchese et al., 2017).

Several studies indicate that eugenol facilitates ATP and K^+^ ion efflux from bacterial cells. Due to its hydrophobic nature, eugenol easily penetrates the outer membrane of Gram-negative bacteria, integrates into the phospholipid bilayer, and disrupts membrane integrity. Additionally, its membrane-permeabilizing ability suggests potential synergy with antibiotics (Zhang et al., 2017). Clove oil affects cell permeability by reducing the activity of enzymes such as *β*-galactosidase and alkaline phosphatase (AKP) and inducing leakage of biological macromolecules like ATP, proteins, and DNA. Furthermore, clove essential oil disrupts the tricarboxylic acid (TCA) cycle by reducing the activity of key enzymes, including citrate synthase, *α*-ketoglutarate dehydrogenase, and isocitrate dehydrogenase. Most importantly, eugenol alters DNA conformation by forming a eugenol-DNA complex (Cui et al., 2018b).

The molecular mechanism of cloves essential oil has been explored in *E. coli* and *S. aureus*. The MIC and MBC values of eugenol were 0.32 mg/mL and 0.64 mg/mL for *E. coli* and 0.26 mg/mL and 0.52 mg/mL for *S. aureus*, respectively. Additionally, eugenol exhibited antibiofilm activity at 4 × MIC, with inhibition rates of 88.27% for *S. aureus* and 73.12% for *E. coli*, indicating a concentration-dependent disruption of biofilms. Furthermore, incubation of *S. aureus* with eugenol led to increased production of reactive oxygen species (ROS), hyperactivation of antioxidant enzymes such as superoxide dismutase, glutathione peroxidase, and catalase, ultimately resulting in oxidative stress-induced cell death (Bai et al., 2023). The surge in ROS leads to lipid peroxidation of the cell membrane and subsequent DNA and protein damage (Sun et al., 2018). At the biochemical level these events form a causal chain: hydrophobic eugenol first partitions into lipid bilayers, increasing membrane fluidity and permeability and producing rapid K^+^ and ATP leakage with PMF collapse. PMF collapse and reduced ATP production immediately compromise energy-dependent repair, active transport and efflux, permitting greater intracellular access of other phytochemicals. Simultaneously, perturbation of the electron transport chain and TCA-cycle enzymes (e.g., citrate synthase, α-ketoglutarate dehydrogenase) increases electron leakage and ROS formation, driving lipid peroxidation and oxidative damage to proteins and nucleic acids. Importantly, Bayode et al. (2024) frame eugenol as a context-dependent oxidant: at low exposures eugenol may show antioxidant activity, but at high local concentrations (as achieved at the disrupted membrane interface or with concentrated formulations) it behaves as a pro-oxidant that overwhelms bacterial redox homeostasis; thus converting membrane perturbation into sustained oxidative injury and bactericidal stress. The combined outcome is multi-target collapse (membrane failure → metabolic/TCA dysfunction → oxidative injury → macromolecular inhibition) that can overcome individual resistance mechanisms.

Additionally, bacterial antioxidant defense mechanisms attempt to neutralize oxidative stress by upregulating intracellular enzyme activity following eugenol exposure (Lin et al., 2019). Moreover, eugenol disrupts DNA synthesis, preventing bacterial replication and leading to cell death (Cui et al., 2018a). In *Bacillus cereus*, eugenol has also been shown to inhibit the production of enzymes such as amylases and proteases (Liu et al., 2017).

One study detailed the multifaceted antibacterial mechanisms of clove essential oil against *S. aureus*. These include: (i) inhibition of the tricarboxylic acid cycle via suppression of key respiratory enzymes (ii) disruption of DNA replication and transcription through alterations in DNA conformation, and (iii) modulation of the *agr* (accessory gene regulator) quorum-sensing system, a central regulator of bacterial virulence. Specifically, cloves essential oils downregulated the expression of *agrA* and *agrC*, attenuating pathogenicity. Collectively, these findings underscore the potential of clove essential oil as a natural antimicrobial agent capable of mitigating bacterial resistance (Li et al., 2022).

In general, the primary antibacterial mechanism of clove oil involves disrupting the bacterial membrane, leading to cell death (Dua et al., 2014). In summary, clove essential oil’s antibacterial activity follows a three-step mechanism: (i) interaction with the bacterial cell wall and membrane, causing intracellular leakage; (ii) penetration into the cytoplasm and interference with cellular structures; and (iii) inhibition of essential processes such as DNA and protein synthesis, which are critical for bacterial growth and survival (Figure 4). This multifaceted mechanism confirms its efficacy at the molecular level and its potential application as a natural antimicrobial agent (Xu et al., 2016).

### 3.2 Clove buds as a quorum sensing inhibitor

**Figure 5:**
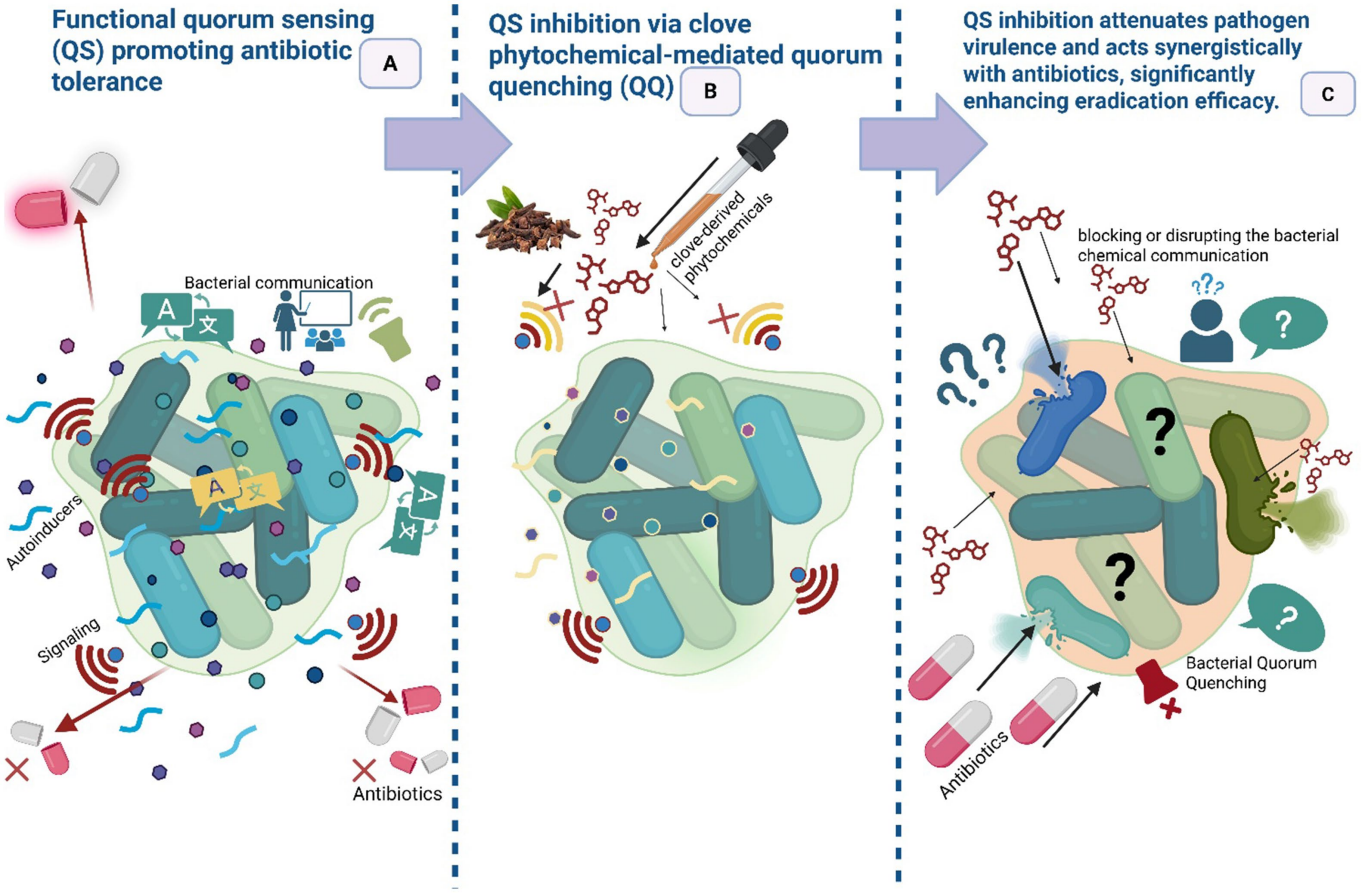
Attenuation of virulence and antibiotic synergy following quorum sensing inhibition (Disarm then treat strategy). **(A)** Functional quorum sensing (QS) promotes virulence and antibiotic tolerance in a bacterial community. **(B)** Clove phytochemicals act as quorum quenching (QQ) agents, disrupting QS to disarm pathogens by inhibiting virulence and tolerance mechanisms. **(C)** This disarming synergizes with conventional antibiotics, leading to significantly enhanced bacterial eradication. (This figure was created by the author using BioRender).

Quorum sensing (QS) is a sophisticated cell–cell communication process wherein bacteria collectively monitor their population density by producing, secreting, and detecting the accumulation of specific chemical signal molecules, known as autoinducers, in their extracellular environment. As a bacterial community proliferates, the increasing concentration of these autoinducers serves as a metric for cell density, ultimately triggering a coordinated shift in gene expression that regulates community-wide behaviors (Federle and Bassler, 2003). Biofilm formation arises from bacterial aggregation, where increasing cell density triggers the release of chemical signals that mediate communication through the QS system (Putri et al., 2025). QS interference is an important anti-virulence mechanism by which clove phytochemicals can “disarm” pathogens without applying direct lethal pressure rather than killing cells, QS inhibitors attenuate collective behaviors that mediate virulence, biofilm formation, toxin production, and coordinated resistance mechanisms, and therefore may impose less selective pressure for conventional resistance (Figure 5). Lawani et al. (2023) framed QS inhibition as a physicochemically informed anti-virulence strategy: small lipophilic phytophenols (e.g., eugenol) can perturb membrane microdomains and disrupt signal transduction or ligand–receptor interactions at the cell surface, thereby reducing QS signaling amplitude and downstream virulence gene expression. Several studies on clove and eugenol report downregulation of QS-regulated genes and reduced biofilm biomass at sub-inhibitory concentrations (Husain et al., 2013; Al-Shabib et al., 2017). An interesting study revealed that clove bud extracts (hexane, chloroform, and methanol) exhibit multi-target anti-quorum sensing activity. Hexane and methanol suppressed violacein production in *Chromobacterium violaceum*, chloroform and methanol reduced *E. coli* bioluminescence, and in *Pseudomonas aeruginosa*, the extracts inhibited multiple QS-regulated traits, including *lecA:lux* expression (hexane), swarming (methanol), and pyocyanin production (hexane) (Krishnan et al., 2012). Therefore, quorum-quenching should be viewed as a complement to bactericidal strategies, a strategic “disarm, then treat” approach that may slow resistance evolution.

In nature, quorum quenching (QQ) represents a natural competitive strategy in which bacteria and other organisms secrete enzymes such as lactonases and acylases to degrade quorum-sensing signals. By disrupting intercellular communication, these enzymes suppress virulence and biofilm formation in competing species, conferring ecological advantage without exerting direct bactericidal pressure (Sikdar and Elias, 2020). Enzymes involved in quorum quenching, including lactonases and acylases, disrupt conserved features of signaling molecules, thereby interfering at several stages of the quorum-sensing process. Such multi-target activity greatly limits the potential for resistance, as it would demand parallel modifications in signal generation, export, and detection of an evolutionarily demanding outcome (García-Contreras et al., 2013). This “disarm-then-treat” strategy may prove highly beneficial, particularly when quorum-quenching is combined with clove-derived antibacterials such as eugenol. Such synergism holds the potential to restore the efficacy of conventional antibiotics compromised by QS-mediated resistance, highlighting a critical research gap that warrants further investigation.

### 3.3 Synergistic effects with conventional antibiotics

The concept of synergistic selectivity in plant extracts describes how multiple phytochemicals act cooperatively to produce a stronger antibacterial effect than individual constituents alone, frequently demonstrating enhanced specificity against particular bacterial species while maintaining comparatively low toxicity to patient (Stefanović, 2017). Unlike conventional antibiotics that target single cellular mechanisms, plant-derived antimicrobials act through synergistic interactions among diverse bioactive compounds. Single-target antibiotics exert specific selection pressures that bacteria often avoid by route mutation, while complex phytochemical combinations, such as clove essential oil, simultaneously affect several cellular targets (Burt, 2004). This multi-target strategy disrupts microbial survival by various mechanisms (Figure 4). Inspired by this natural defense system, researchers have enhanced antibiotic efficacy by combining plant phytochemicals with synthetic efflux pump inhibitors, demonstrating the potential of phytochemical-based antimicrobial strategies (Hemaiswarya et al., 2008).

**Figure 6:**
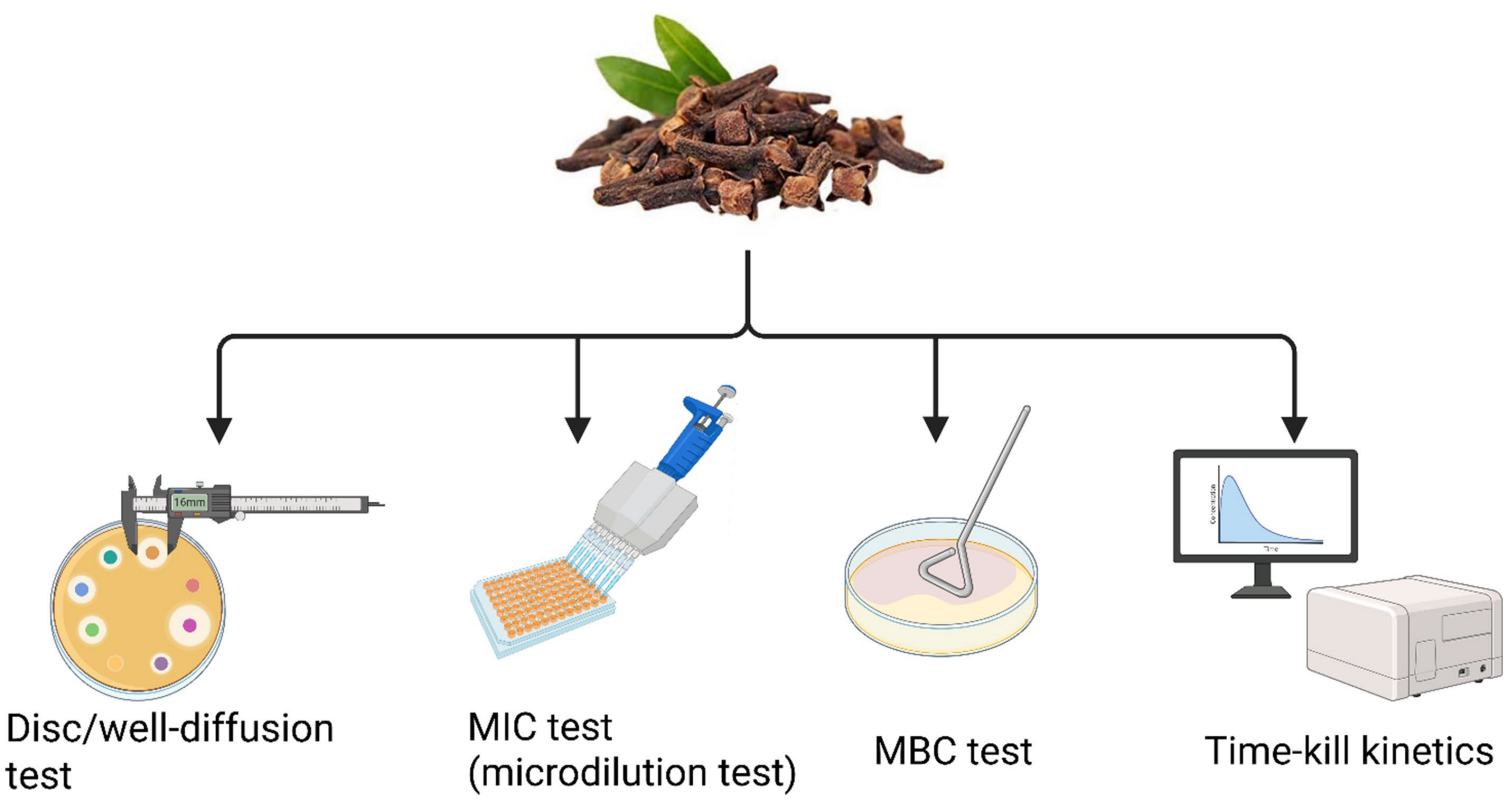
Overview of major *in vitro* antibacterial assays of clove buds against MDR bacteria. This schematic figure provides a conceptual roadmap of the key methodologies employed to rigorously evaluate the antibacterial efficacy of *S. aromaticum* extracts against MDR bacterial pathogens. The figure guides the researcher from initial, high-throughput screening assays to more definitive, quantitative measures of bactericidal activity, illustrating a comprehensive workflow for natural product discovery. (This figure was created by the author using BioRender).

In literature, numerous studies have demonstrated that plant-derived molecules (including cloves) enhance antibiotic efficacy by circumventing resistance mechanisms, even in the absence of intrinsic antimicrobial activity. This synergistic interaction not only potentiates antibacterial effects but also allows for lower drug dosages, thereby reducing toxicity and adverse side effects (Aiyegoro and Okoh, 2009). Scientific literature indicates that the most commonly used methodologies to evaluate the antibacterial activity of cloves against MDR bacteria include disc/well diffusion, MIC/MBC determination, time-kill kinetics, and the checkerboard assay (Figure 6). Although the disk diffusion method is faster, it lacks quantitative precision. In contrast, the broth microdilution technique yields accurate MIC values and is generally preferred, as the results between the two methods often show poor correlation (Liu et al., 2012).

Several studies have reported significant synergistic effects between clove-derived compounds and conventional antibiotics, enhancing their antibacterial efficacy. As shown in (Table 1), these interactions have demonstrated potential in overcoming resistance mechanisms, making clove a promising adjunct in antimicrobial therapy. Table 1 includes Fractional Inhibitory Concentration indices (FICI) derived from checkerboard assays in the original studies. FICI values quantify interaction types (≤0.5 = synergy). Where original papers did not report a full FICI. Therefore, we computed only the antibiotic fractional inhibitory concentration (FIC) contributions: (FIC = MIC of antibiotic in combo ÷ MIC of antibiotic alone) for rows where both MIC values were reported. Several antibiotic contributions are markedly reduced (for example, colistin FICI: 0.0625–0.125 and multiple *β*-lactam entries ≤0.25), indicating large antibiotic MIC-fold reductions when combined with clove-derived agents, a finding that is consistent with synergy when the partner (phytochemicals of clove) contribution is also small. Therefore, it is recommended that synergy studies should calculate the fractional inhibitory concentration index (FICI), defined as:


$$\begin{array}{l} \text{FICI}={\mathrm{FIC}}_{A}+{\mathrm{FIC}}_{B}=({\mathrm{MIC}}_{A\;\text{in combination}}/{\mathrm{MIC}}_{A\;\text{alone}})\\ +({\mathrm{MIC}}_{B\;\text{in combination}}/{\mathrm{MIC}}_{B\;\text{alone}}), \end{array}$$

**Table 1:** Examples of the combined antimicrobial efficacy of clove-derived bioactive compounds and antibiotics.

Bacterial pathogen	Antibiotic exhibits synergism with clove	MIC of antibiotic alone (μg/mL)	MIC of Combination (μg/mL)	FIC index* (μg/mL)	References
*Streptococcus mutans*	Ampicillin	2.0	0.5	0.250	Moon et al. (2011)
*S. mutans*	Gentamicin	8.0	2.0	0.250	Moon et al. (2011)
*Pseudomonas* sp.	Imipenem Amoxcillim+clavulanic acid	1.95 1,000	0.48 7.81	0.246 0.0078	Al-Tawalbeh et al. (2025)
*S. aureus* *S. epidermidis*	Vancomycin	1,024	512 128	0.500 0.125	Jafri and Ahmad (2021)
*S. mutans*	Azithromycin	128	32	0.250	Jafri and Ahmad (2021)
*E. coli*	Nitrofurantoin Ciprofloxacin	64 1,024	32 512	0.5 0.5	Marouf et al. (2023)
*Acinetobacter* sp. *Klebsiella* sp.	Colistin	64 64	4 8	0.0625 0.125	Vázquez-Ucha et al. (2020)
*Klebsiella* sp.	Ceftriaxone Ceftazidime Cefepime	100 12.5 50	12.5 3.12 3.12	0.125 0.249 0.062	Ibrahim et al. (2019)
*S. aureus*	Ciprofloxacin	256	64	0.256	Abou El Nour et al. (2018)
*E. coli* *S. aureus*	Ampicillin Amoxicillin+ clavulanic acid Amoxicillin+ clavulanic acid Oxacillin	90 100 90 70	17.14 8.5 25.7 8.5	0.190 0.085 0.285 0.121	Elamary et al. (2023)
*Salmonella typhimurium* *S. enteritidis*	Oxytetracycline	512 256	64 128	0.125 0.250	Huerta Lorenzo et al. (2024)
*S. aureus*	Cefotaxime	0.0024	0.0002	0.0833	Lady et al. (2023)

*FIC index: Fractional Inhibitory Concentration index; FIC values in this table were calculated by the authors from the MICs shown in the table from the cited references (FIC = MIC in combination ÷ MIC alone). FIC ≤ 0.5 indicates synergy, meaning the antibiotic and phytochemical inhibit bacteria far more effectively together than alone.

where “A” is the conventional antibiotic and “B” is the clove-derived phytochemical (or essential oil constituent). FICI values ≤ 0.5 indicate synergy (Wambaugh et al., 2019).

However, these computed antibiotic-only FICs (Table 1) are partial measures: a valid interpretation of synergy requires the phytochemical’s FIC (FIC-B) or the full checkerboard FIC index (FICI = FIC-A + FIC-B). The FICI threshold convention (≤0.5 = synergy) is widely used but has known limitations and can be misleading without standardized methods and confirmation by orthogonal assays (e.g., time-kill) (Odds, 2003). Also, because checkerboard-derived FICIs can vary with method (inoculum, endpoint, extract standardization) and may not fully predict *in vivo* interactions, we recommend completing FICI calculations by retrieving partner-MICs (or performing two-dimensional checkerboards) and confirming promising combinations with time-kill studies or PK/PD modeling prior to claiming clinical-relevant synergy (Bentley, 2024). Notably, some of the source studies in Table 1 did provide full FICI indices using checkerboard assays (Not mentioned in the table as most studies have no FICI). Jafri and Ahmad (2021) demonstrated strong synergy between eugenol and azithromycin (FICI = 0.141) as well as between clove oil and vancomycin (FICI range 0.25–0.50) against *Staphylococcus* spp. and *S. mutans*. Vázquez-Ucha et al. (2020) reported FICI ≤ 0.5 for clove oil–colistin combinations against MDR *Acinetobacter* and *Klebsiella*, confirming synergy in clinically challenging strains. Huerta Lorenzo et al. (2024) observed additive to borderline synergistic interactions for clove oil with oxytetracycline against *Salmonella* (FICI 0.625–1), highlighting strain-specific variability. Similarly, Ibrahim et al. (2019) reported FICI < 0.5 for clove volatile oil combined with *β*-lactams (ceftriaxone, ceftazidime, cefepime) against *Klebsiella pneumoniae*. These examples underscore that full FICI determinations are feasible and can robustly confirm synergistic activity, while also illustrating the diversity of interaction outcomes depending on strain, antibiotic partner, and phytochemical preparation.

It was reported that eugenol exhibits antibacterial activity against cariogenic and periodontopathogenic bacteria. Time-kill studies confirmed its synergy with ampicillin and gentamicin, showing a significantly higher reduction in CFU/mL after 60 min of treatment compared to individual agents (Moon et al., 2011). The combination of clove extract with imipenem and amoxicillin-clavulanic acid demonstrated significant synergistic effects, leading to a notable reduction in the minimum inhibitory concentrations (MICs) of these antibiotics against *P. aeruginosa* (Al-Tawalbeh et al., 2025). The antibacterial and antibiofilm properties of eugenol extracted from clove oil were demonstrated through its ability to enhance antibiotic efficacy and disrupt bacterial biofilms. At sub-inhibitory concentrations, reductions in bacterial surface hydrophobicity and hemolysin production were observed. Unlike conventional antibiotics, which exhibited a 1,000-fold decrease in effectiveness against biofilms, eugenol maintained its potency. Significant synergy with azithromycin was noted, and microscopy confirmed the disruption of mature *S. aureus* and *S. mutans* biofilms, highlighting the potential of clove-derived compounds in combating resistant bacterial biofilm (Jafri and Ahmad, 2021). Clove extract exhibited concentration-dependent antibacterial activity against uropathogenic *E. coli* and *E. coli* ATCC 25922, with MIC/MBC values of 25 mg/mL and 6.25/25 mg/mL, respectively, confirming its bactericidal effect. Although no formal synergy was detected with nitrofurantoin or ciprofloxacin, MIC reductions were observed in combination treatments. Notably, clove extract induced 96–99% of uropathogenic *E. coli* to transition into unstable spherical L-forms, indicating potential cell wall disruption (Marouf et al., 2023). Clove and thyme essential oils (EOs) demonstrated potent synergy with colistin against MDR *Acinetobacter baumannii* and *K. pneumoniae*. In resistant strains, combining essential oils (EOs) with colistin reduced colistin’s MIC by 8–128-fold, with similar effects in susceptible strains. Time-kill assays confirmed enhanced bactericidal activity. These EO-antibiotic combinations enable lower, safer colistin doses while overcoming resistance, offering a promising strategy to revitalize colistin efficacy against critical pathogens (Vázquez-Ucha et al., 2020). Clove volatile oil exhibited strong antibacterial activity and inhibited AmpC *β*-lactamase, enhancing susceptibility in *K. pneumoniae* isolates prevalent in Al Anbar hospitals, Iraq. Its synergy with third- and fourth-generation cephalosporins (ceftazidime, cefepime, ceftriaxone) significantly improved bacterial response, highlighting its potential as a natural adjuvant to restore antibiotic efficacy against AmpC-producing strains. Molecular identification of *AmpC* genes remains essential for monitoring hospital transmission, reinforcing the role of clove oil in combating MDR infections (Ibrahim et al., 2019). Clove extract and ciprofloxacin exhibited high MICs against MDR *S. aureus* (1,024 and 256 μg/mL, respectively). However, their synergistic combination significantly enhanced antibacterial efficacy, effectively overcoming resistance. These findings suggest that clove extract could enhance ciprofloxacin’s potency, allowing for reduced antibiotic doses in the treatment of MDR pathogens (Abou El Nour et al., 2018). The combination of oxytetracycline and clove essential oil exhibited additive effects (FIC index = 0.625–1) against *Salmonella* spp. Clove essential oil reduced the required concentrations of both oxytetracycline (by 2–8×) and itself (by 2–4×), effectively lowering therapeutic doses while maintaining antibacterial efficacy (Huerta Lorenzo et al., 2024). Eugenol purified from clove buds synergized with cefotaxime against *S. aureus* strains (ATCC 33591, 29213, 25923), including MRSA variants. This combination enhances cefotaxime’s efficacy, suggesting its potential to revitalize β-lactam antibiotics against resistant infections (Lady et al., 2023).

## 4 Efficacy against MDR pathogens

The U. S. Centers for Disease Control and Prevention (CDC) estimated approximately 2.8 million antibiotic-resistant infections, resulting in about 35,000 deaths annually (Guardabassi et al., 2020). Extensive inappropriate use and misuse of antibiotics further exacerbate this problem, accelerating the selection and spread of antimicrobial resistance (Christaki et al., 2020). On account of this, there is increasing demand to explore alternative strategies to prevent and control bacterial infections. Among the most promising are plant-derived compounds, notably polyphenols, alkaloids, and tannins, which can modulate mechanisms of antimicrobial resistance (AlSheikh et al., 2020). These plant-derived compounds have also been shown to potentiate conventional antibiotics against MDR pathogens (Demgne et al., 2021).

Cloves essential oils in previous studies found to be exhibiting killing effect on both gram positive and gram negative bacteria, cloves essential oil inhibited the growth of two β-lactamase producing bacteria including *E.coli* and *K. pneumonia* (Ginting et al., 2021). Additionally also inhibited other gram negative uropathogens which were including *E.coli, Klebsiella* sp., *Enterobacter* sp., *Citrobacter* sp., *Proteus* sp., *P. aeruginosa*, *A. baumannii* all of which are *AmpC* beta-lactamase, metallo beta-lactamase, and extended spectrum beta-lactamases (Faujdar et al., 2020). Cloves nanoemulsion also impart antimicrobial properties on *S. aureus* (Nirmala et al., 2019). Cloves antibacterial effect and its contribution on *Mmr* efflux pump through molecular docking experiments against *Mycobacterium tuberculosis* was accessed which revealed that its susceptible in range of 10 and 100 μg/mL and its component eugenol bid strongly with *Mmr* EP proteins (El Ghallab et al., 2023).

It has been reported that the combination of clove extract with certain antibiotics, including imipenem and amoxicillin-clavulanic acid, results in a significant reduction in their respective MICs, suggesting its potential as an antibacterial agent capable of lowering the required dosages of existing antibiotics (Al-Tawalbeh et al., 2025). Another study evaluated the potential of cloves extract against MDR uropathogens, involve in causing UTI, the diethyl ether extract of clove marked big inhibition zones against *S. aureus* (19 mm) and *K. pneumonia* (19 mm) and less against *Pseudomonas* sp. (11 mm) and *E. coli* (12 mm) with highest concentration at 200 mg/mL and lower at 6.25 mg/mL while the ethanolic extract of clove at concentration of 200 mg/mL impart inhibition zones of 18 mm for both *S. aureus* and *K. pneumonia* but no inhibition was observes at 6.25–12.5 mg/mL against *E. coli*, *P. aeruginosa*, and *Enterobacter* spp. (Salisu et al., 2021). Cloves also represent highly effective against WHO priority list pathogens *A.baumannii* and *Klebsiella pneumonia* when combined with last resort drug called colistin lower the MIC by 8–64-folds and 8–128-folds, respectively, (Vázquez-Ucha et al., 2020). Antibacterial activity of clove oil at concentration of 10–2 μL/mL observed against MDR *Pseudomonas* sp. and *Burkholderia cepacia* complex both of which isolated from burn infections and intensive care unit (Mostafa et al., 2017). A study investigated the activity of clove against zoonotic MDR pathogen *Streptococcus suis*, at 15% concentration maximum zone of inhibition was observed plus the time kill assay revealed that within 15 min of exposure completely reduced this pathogen urging its use as alternative option for prevention of infectious diseases both in animals and humans (Wongsawan et al., 2019).

*Pseudomonas* sp. isolated from canine otitis is considered a significant public health concern, having been classified as a critical pathogen by the WHO. Susceptibility of these isolates was preserved using clove essential oil, with MIC and MBC values determined to range from 3.26 to 6.53 mg/mL, and the primary activity being attributed to eugenol (Costa et al., 2022). A study in Egypt conducted on UTI, in which from urine samples most dominant pathogen was *Pseudomonas* sp. containing antibiotic resistant genes such as *blaTEM*, *blaSHV*, etc. against which ethanolic extract of clove showed inhibition zone of 23 mm and MIC and MBC range from 10 to 121.25 mg/mL and 20 to 30 mg/mL, respectively, presenting cloves as potential agent to treat MDR bacteria (Ahmed et al., 2021). The anti-virulence and antibiofilm potential of clove bioactive fraction was evaluated against *Pseudomonas* sp. from catheter associated UTI, eugenol in CBF lower the expression of mRNA levels of quorum sensing receptor genes at the concentration of 700 μg/mL (Rathinam and Viswanathan, 2018). Previously a study on corneal ulcer showed that main MDR pathogens are commonly involve in this disease and most common one is *S. aureus* in all cases, for which the activity of cloves found to be effective upon demonstration of its highest sensitivity rate 97.5% along with low MIC value 0.10 μL/mL, explicitly leveling up the usage of cloves treatment of ocular bacterial infections (Mohamed et al., 2018). Clove also exhibit activity against other urinary tract infections caused by *E.coli* and *K. peumoniae*, with 16 mm inhibition zone for *E. coli* and 12 mm for *klebsiella* sp. while the MIC of them was 0.55 μL/mL (Băicuș et al., 2022). Clove oil also imparts inhibitory effects on plasmid mediated AmpC *β*-lactamase enzymes producing genes frequently isolated from *Klebsiella* sp. as an important MDR nosocomial pathogens responsible for major morbidity and mortality, along within also harbor synergistic effect toward β-lactam antibiotics effectively urging the fact that clove oil can helpful in improving their susceptibility profiles (Ibrahim et al., 2019). The potential of clove oil against the virulence of *Klebsiella* sp. was determined, the main virulence caused by capsule formation, a study in Egypt explored that clove oil can act as anti-virulent agent in destroying the capsule structure or reduce their size and also as promising agent for resistance modification (Edward et al., 2020). The effect of clove oil on MDR isolates of *E. coli* and *K. pneumoniae* obtained from patients with chronic hepatic disease was assessed. Treatment with clove oil resulted in a significant reduction in the colony counts of both pathogens compared to pre-treatment levels. The MICs of clove oil were determined to be 0.04 mL for *E. coli* and 0.05 mL for *K. pneumoniae*, with corresponding inhibition percentages of 11 and 9%, respectively. These findings suggest that clove oil may serve as a potential adjunct therapy to mitigate secondary bacterial infections in patients with chronic liver disease (Shaaban et al., 2024). Another study explored the antibacterial potential of cloves against four pathogenic bacteria including *S. aureus*, MRSA, *E. coli*, and *S. typhi*; the dichloromethane extract of clove oil form the inhibition zones of 18.20, 17.25, 21.15, and 24.2 mm, respectively, while the MIC of 1 mg/disc for *S. aureus,* and MRSA and 0.5 mg/disc for *E.coli* and *S. typhi* thus maintaining the trend of highest sensitivity of clove oil against gram negative isolates as compare to gram positive isolates (Yassin et al., 2020). The virulence factor quorum sensing and biofilm formation are also been investigated for their control in *Pseudomonas* sp. and *Aeromonas hydrophilia*, it has been seen that clove oil at sub inhibitory concentrations lower the virulence agents such as LasB, total proteases, pyocyanin production, chitinase, exoploysaccharides production and swimming motility on the other hand reduce the biofilm formation ability of *Aeromonas* sp. in concentration dependent manner (Husain et al., 2013). Just like this one more research was conducted on *Pseudomonas* sp. for quorum sensing and virulence aspect with addition of efflux pump encoding genes (*mexA* and *mexB*) all of them reduce to significant level when treated with clove oil at ½ MIC (El-Banna et al., 2023). Clove oil efficacy also being observed against wound pathogen MRSA when it lower the microbial load present in wounds and can to high extent produce good antibacterial effects alone or in combination (Alanazi et al., 2022). One of the recent studies evaluate the clove potential under different conditions in room temperature sunlight and low temperature dark, the extract of cloves prepared in dark temperature exhibit big inhibition zones with diameter of 13, 20, 20, 21, and 15 mm against *S. aureus, S. epidermidis, P. aeruginosa, K. pneumonia,* and *E. coli*. Whereas extract of clove in light room temperature exhibited zones of inhibition 17,10, 15, 18, 17 mm, respectively (Mekky et al., 2024). Similarly, one study reported the examination of natural products particularly cloves as potent therapy for threat of MDR bacteria among which *K. pneumonia* and *S. aureus* and the calculated MIC for *K. pneumonia* was 12.5 mg/mL which also emphasize the use of cloves to overcome this main problem (Mohamed et al., 2020). Another study investigated the role of clove essential oil against MDR *P. aeruginosa* and its virulence genes particularly of biofilm and protease production, upon RT-PCR showed that clove oil at sub inhibitory MIC concentration lower the expression levels of virulence genes suggesting its use for treatment of MDR bacteria (Awad et al., 2024). One of serious problem causing pathogen *Enterococcus faecalis* isolated frequently from women suffering from urinary tract infections on which the antimicrobial effect of cloves were determined with diameter of inhibition zones obtained in the range of 21.13–7.14 mm suggesting it as novel alternative for UTIs problem of resistance (Saleh et al., 2024). Study from Pakistan for profiling of antibacterial and antibiofilm aspect of clove against multidrug resistant human pathogens was done, in which different extracts of clove were prepared to check the inhibitory effect against *S. saprophyticus*, *K. pneumonia, S. pyogenes*, *E. coli*, *S. aureus*, and *P. aeruginosa*, the MIC values of clove oil extract were 40, 80, and 160 μg/mL regarding its antibacterial, antibiofilm and antioxidant properties highlighting the importance of secondary metabolites as source of fight against resistance (Fatima et al., 2023).

To assess the comparative antimicrobial efficacy, several studies have investigated the activity of clove oil and other natural agents against MDR pathogens. One study evaluated the antimicrobial potential of clove and rosemary oils against four MDR bacteria, S*. aureus*, *Enterococcus faecalis*, *P. aeruginosa*, and *A. baumannii*. The minimum inhibitory concentration (MIC) values of rosemary oil ranged from 0.312 to 5%, whereas clove oil demonstrated MICs between 0.312 and 1.25%, indicating superior inhibitory activity of clove oil over rosemary oil, supporting its use as a natural antimicrobial agent (Abdullah et al., 2015). Another investigation assessed the antibacterial activity of ethanolic and aqueous extracts of clove, cinnamon, and garlic against *E. coli* isolates from urinary tract infections (UTIs). Clove exhibited the highest antibacterial effect with a mean inhibition zone of 13.33 mm, compared to 11.33 mm for cinnamon. Ethanolic extracts of cinnamon and garlic produced inhibition zones of 14 mm and 16 mm, respectively, whereas clove oil generated the largest zone of inhibition at 27 mm (Noreen et al., 2018). In a comparative study against *E. faecalis*, clove and thyme were evaluated for their antibacterial potential. The aqueous phase of thyme showed no inhibitory effect, with a mean zone of 0 mm, while clove produced a mean inhibition zone of 13.67 mm (Agarwal and Yeluri, 2023). Additional research on the antibacterial properties of aqueous and ethanolic extracts of clove and garlic against *Staphylococcus* sp., *Streptococcus* sp., *Pseudomonas* sp., *Klebsiella* sp., and *E. coli* revealed that ethanolic clove extract had the highest inhibitory effect, followed by the aqueous extract. Garlic extracts exhibited moderate to low antibacterial activity. Clove showed inhibition zones up to 26 mm against *Klebsiella* sp. and 20 mm against *Staphylococcus* sp., with MIC values ranging from 64 to 128 μg/mL across pathogens (Liu et al., 2021). In another comparative study, several plant-based agents, including clove, turmeric, cinnamon, nutmeg, and peppermint oils, were assessed for their antimicrobial activity against *E. faecalis* and *S. mutans*. Clove oil exhibited the most potent activity, with inhibition zones of 29.8 and 40.33 mm at 100 μL, respectively (Mohapatra et al., 2023). Moreover, a study evaluating the effect of clove oil and licorice on gene expression in *S. mutans*, a key agent in dental caries, revealed that clove oil significantly downregulated the expression of virulence genes *gtfB* and *gtfD*, with fold changes of 0.178, 0.454, and 0.191 compared to licorice extract, based on RT-PCR analysis (Al-Amili and Al-Jobori, 2025). Lastly, the antibacterial and antioxidant activities of clove pollen grain extract were compared with maize extract against *Salmonella* sp.*, E. coli, and S. aureus*. Clove extract demonstrated higher sensitivity, with inhibition zones of 29, 18, and 22 mm, respectively, and also exhibited stronger antioxidant capacity (Barnawi et al., 2023).

## 5 Pharmacokinetics and bioavailability of clove compounds

**Figure 7:**
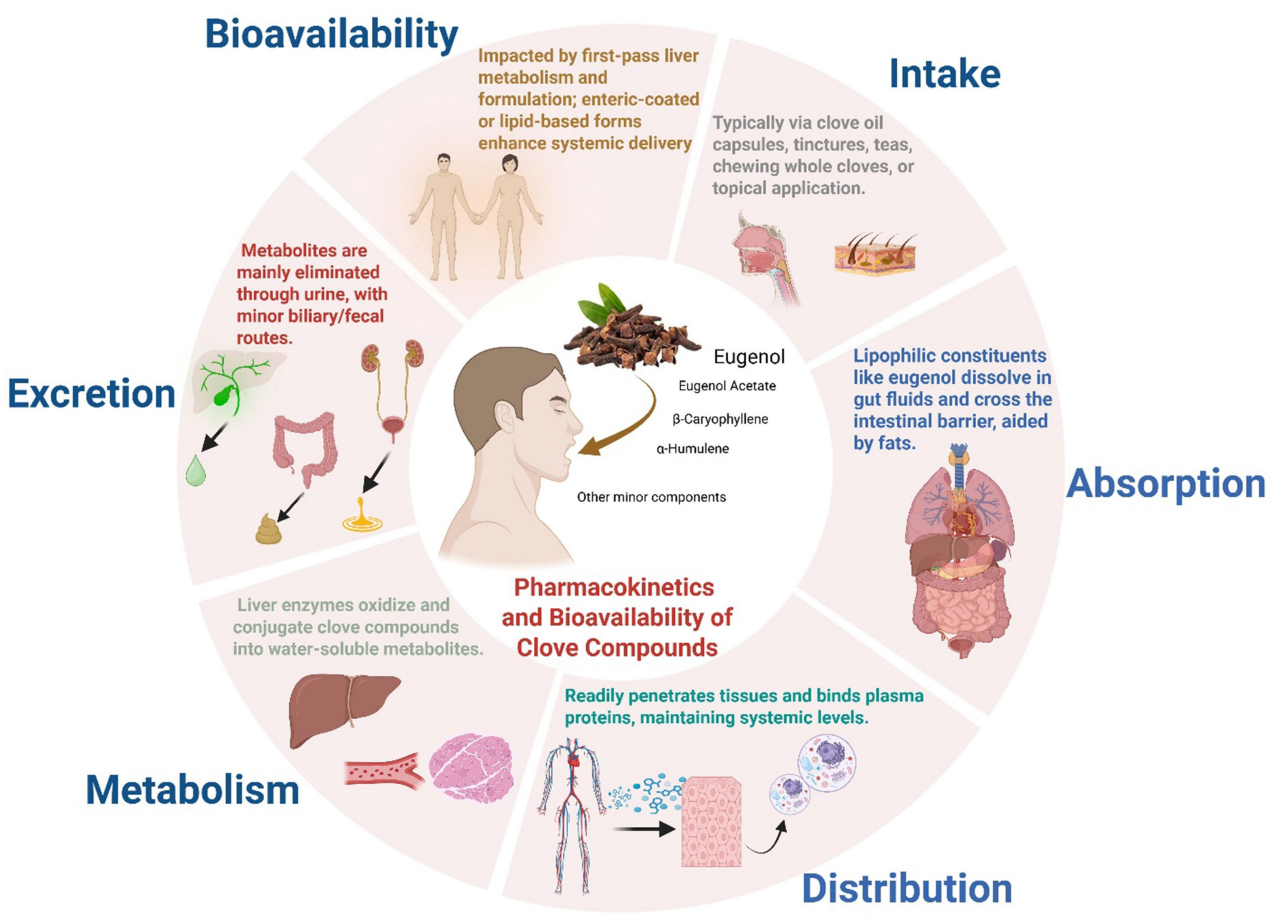
Possible pharmacokinetics and bioavailability of clove compounds. This figure provides schematic overview of the ADME pathway of clove bioactives (e.g., eugenol, β-caryophyllene), illustrating their pharmacokinetic journey from intake through absorption, metabolism, and elimination, and emphasizing how these processes govern systemic bioavailability and *in vivo* therapeutic potential. (This figure was created by the authors using BioRender).

Pharmacokinetics is a dynamic, multidisciplinary field that converts intricate biological processes into quantitative models and expressions. By synthesizing ideas from chemistry, biology, and physiology, it is essential across the pharmaceutical continuum from drug development to the clinical refinement of dosage methods for illness treatment or cure (Figure 7). A thorough comprehension of medication physicochemical properties and human physiological processes is crucial for precisely forecasting and assessing therapeutic and clinical results (Ghazi and Cawley, 2021). In scientific literature. The pharmacokinetic profile of clove oil constituents has been investigated only modestly. It was reported that, eugenol, the major phenolic component, is rapidly absorbed and extensively metabolized after oral intake; in healthy human adults, nearly all of an oral dose is eliminated in urine within 24 h, predominantly (>90%) as glucuronide and sulfate conjugates (Nejad et al., 2017). In rodents, eugenol exhibits a relatively long apparent elimination half-life (~14–18 h) (Guénette et al., 2007), Systemic clearance of Eugenol extracted from cloves in male Sprague–Dawley rats averaged 157 mL/min/kg in plasma and 204 mL/min/kg in whole blood. Analysis of urine revealed both glucuronide and sulfate eugenol conjugates (Guenette et al., 2006), consistent with rapid biotransformation. Other major clove constituents show similarly limited bioavailability: for example, *β*-caryophyllene extracted from clove essential oil has poor oral absorption (about 2% bioavailability in rats) (Spigarelli et al., 2024). The interaction varied with lipid type, showing greater monolayer expansion for DPPS (1,2-dipalmitoyl-sn-glycero-3-phospho-L-serine) and DODAB (dioctadecyldimethylammonium bromide), and stronger effects on polar headgroups for DPPS and DPPC (1,2-dipalmitoyl-sn-glycero-3-phosphocholine). These results suggest that eugenol’s ability to interact with and penetrate lipid monolayers of cell depends on lipid composition, supporting its potential to cross biological membranes (Goncalves et al., 2015).

These results suggest that systemic levels of clove compounds after ingestion are generally low unless delivered by specialized carriers. Safety studies indicate that clove oil/eugenol is well tolerated at low concentrations. The U. S. FDA recognizes clove oil as a safe flavoring and cosmetic additive, it is also widely used as dental analgesic (Nejad et al., 2017). However, eugenol is irritant and potentially toxic at high doses as previously reported using animal models (Singletary, 2014). Human case reports note that therapeutic exposures of eugenol have not caused liver enzyme elevations or frank hepatotoxicity, but overdose can produce severe liver injury (National Institute of Diabetes and Digestive and Kidney Diseases, 2012). Clinically, excessive clove oil (undiluted) is known to cause mucosal irritation and systemic symptoms, and “excessive dose” eugenol exposure is judged toxic (Nejad et al., 2017). Thus, while low-dose use (e.g., flavoring) is generally safe, and beneficial for health (Ullah et al., 2023), targeted antimicrobial applications must respect eugenol’s narrow therapeutic margin and potential for allergic or irritant effects. In summary, the pharmacokinetic data indicate rapid absorption and clearance with extensive conjugation (Marchese et al., 2017; Ulanowska and Olas, 2021). These constraints underscore the need for advanced delivery systems and careful dose optimization in any clinical use of clove-derived antimicrobials. Regretfully, data in human patients are still very limited. Future clinical research; for example, trials of clove-derived formulations *in vivo* against specific pathogen or for wound care or as adjunctive oral decontamination, will be needed to validate these preliminary findings.

## 6 Clinical investigations on clove molecules against bacteria

Despite extensive *in vitro* data, few *in vivo* studies and human trials of clove oil in infection exist. Clinical studies have mostly examined clove-containing herbal mouthwashes or topical rinses rather than therapeutic antibiotic use. For example, a randomized, triple-blind ICU trial found that nursing care with a clove-oil–based oral rinse halved the incidence of ventilator-associated pneumonia (VAP) compared to control (20.2% vs. 41.7%) (Jahanshir et al., 2023). The clove group’s VAP risk was roughly 2-fold lower, suggesting a real antimicrobial benefit. Similarly, a controlled trial in dental patients showed that a multi-herbal mouth rinse containing clove extract (with tea tree and basil) produced significant reductions in gingival plaque and microbial colony counts, comparable to a commercial essential-oil mouthwash (Kothiwale et al., 2014). These findings illustrate that clove oil can exert measurable antimicrobial effects *in vivo* when formulated as a rinse or topical antiseptic. The *in vivo* translational (preclinical) studies further support these clinical hints. In a rat model of MRSA-infected wounds, topical clove bud oil alone or combined with imipenem had accelerated wound closure and markedly reduced bacterial counts relative to untreated controls (Alanazi et al., 2022). Likewise, purified clove fractions rich in eugenol have been shown to inhibit biofilm formation and virulence gene expression in multidrug-resistant uropathogens (*Pseudomonas* and *Enterobacter* spp.) recovered from catheter-associated urinary infections (Rathinam and Viswanathan, 2018). These studies confirm that clove compounds can function in complex biological environments at subinhibitory doses, although formal clinical trials are lacking. Notably, clove oil also shows potent *in vitro* activity against other resistant pathogens (e.g., drug-resistant *Helicobacter pylori*, foodborne bacteria, etc.) beside its significant anti-inflammatory potential on human erythrocytes (Elbestawy et al., 2023), but preclinical translational studies on humans has not yet been attempted. In summary, the few available clinical and animal studies suggest clove essential oil can reduce pathogenic bacterial load and infection rates when applied topically or orally (Alanazi et al., 2022; Jahanshir et al., 2023). However, clinical data in human patients remain scarce. There is a critical need for future studies, including well-designed clinical trials, to evaluate the efficacy of clove-derived formulations against internal multidrug-resistant (MDR) bacteria and systemic bacterial infections. Addressing this substantial research gap is essential for advancing therapeutic applications of clove bioactive molecules.

## 7 Formulation strategies for clove-based antibacterials

**Figure 8:**
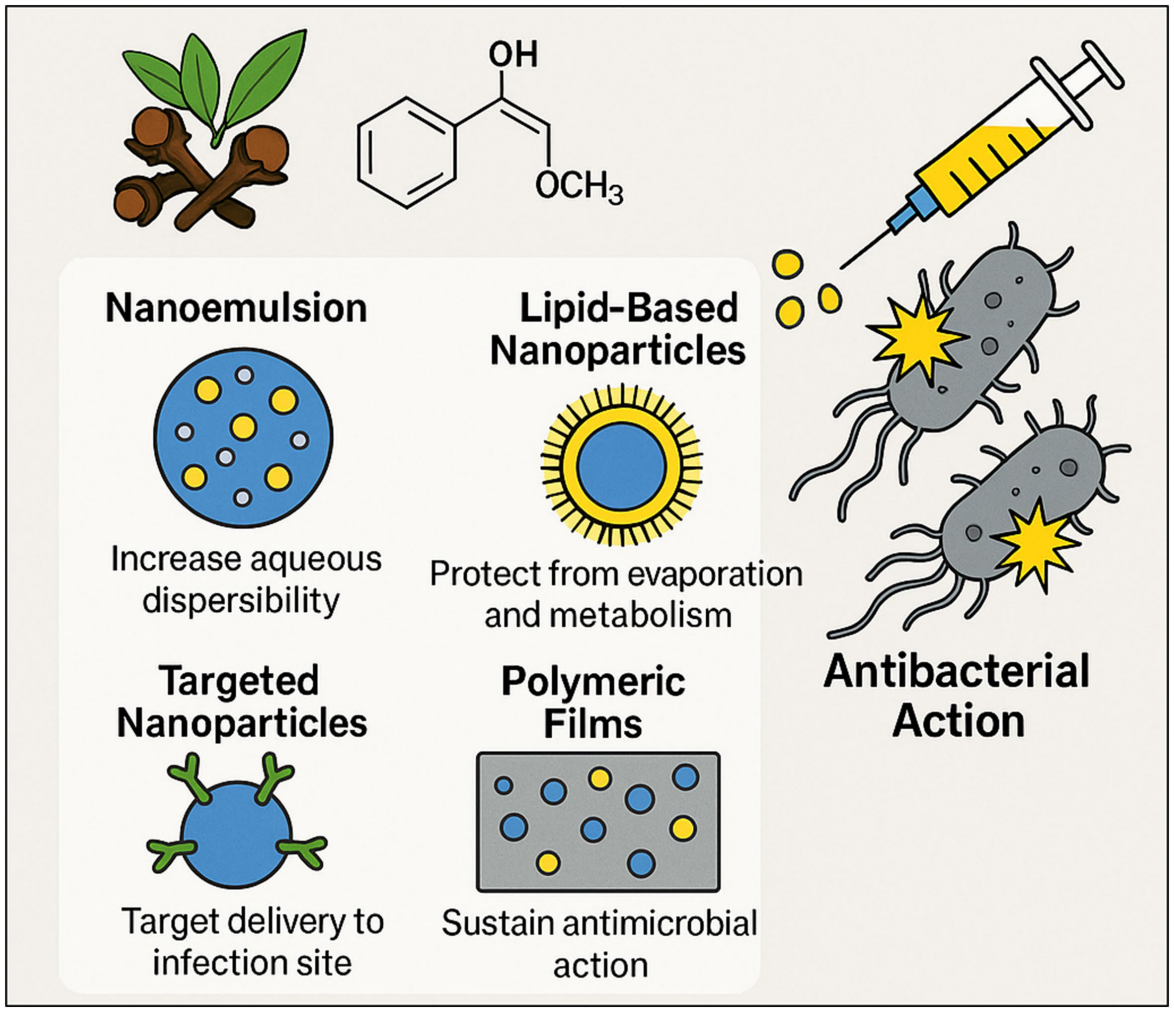
Formulation strategies to optimize antibacterial delivery of clove bioactive molecules. This figure provides a brief conceptual overview of advanced nano- and micro-formulation strategies designed to enhance the stability, bioavailability, and targeted antibacterial delivery of clove bioactives (e.g., eugenol), thereby overcoming physicochemical and pharmacokinetic limitations of raw extracts and improving efficacy against MDR infections. (This figure was created by the authors with the assistance of OpenAI, 2023).

As previously noted, the clinical application of clove-derived bioactive compounds remains limited. Key challenges including volatility, poor aqueous solubility, and metabolic instability necessitate the use of advanced formulation strategies such as nanoemulsions, lipid-based nanoparticles, targeted delivery systems, and polymeric films to enhance their antibacterial efficacy and stability (Figure 8).

Because clove oil components are volatile, hydrophobic, and metabolically labile, many investigators are exploring advanced formulations to enhance their stability, solubility, and antimicrobial efficacy. Nanoemulsions and lipid-based nanoparticles are especially promising (Liñán-Atero et al., 2024; Ye et al., 2024). In one study, a self-emulsifying nanoemulsion of clove oil (droplet size ≈30 nm) exhibited superior antibacterial potency relative to pure oil. The optimal nanoemulsion produced MICs against common pathogens that were comparable to those of a standard antibiotic (amikacin), effectively boosting eugenol’s efficacy (Anwer et al., 2014). Similarly, solid-lipid nanoparticles co-loaded with eugenol and the antibiotic ofloxacin (with chitosan for cationic targeting) achieved striking synergy: the MICs of ofloxacin decreased by 6–16-fold when delivered in these hybrid particles, effectively overcoming resistance in *P. aeruginosa* and *S. aureus* (Rodenak-Kladniew et al., 2019). These lipid carriers also enabled sustained antibiotic release and selective bacterial uptake. Targeted nanoparticle systems have been engineered as well. For instance, antibody-modified liposomes encapsulating clove oil were designed to bind *Campylobacter jejuni* specifically and release eugenol in response to bacterial proteases. This “active” liposome showed greatly enhanced targeting and prolonged antibacterial action against *C. jejuni* on contaminated foods (Chen et al., 2023). Incorporation into polymeric matrices is another strategy: electrospun nanofiber mats (polycaprolactone/gelatin) loaded with clove oil remained highly bactericidal against *S. aureus* and *E. coli*, making them attractive as antibiotic-free wound dressings (Unalan et al., 2019). Likewise, chitosan/PVA films and other biopolymer coatings containing clove oil or eugenol have demonstrated faster and more complete kill kinetics than polymer alone (Kowalewska and Majewska-Smolarek, 2023). Other approaches include solid–solid encapsulation and hybrid nanoparticles. Adsorbing eugenol onto mesoporous silica particles, for example, can “solidify” the oil and accelerate its release without loss of activity (Yao et al., 2024). Inorganic nanomaterials have been employed as well: cloves extract can mediate the green synthesis of silver nanoparticles, and such Ag–eugenol composites show enhanced antimicrobial action. Clove-derived AgNPs exhibited strong antibacterial effects across multiple pathogens and even synergized with clarithromycin (Edis et al., 2025). Finally, all these advanced formulations aim to (1) increase aqueous dispersibility, (2) protect the oil from premature evaporation or metabolism, and (3) target delivery to the infection site. The reported results dramatically lower MICs, sustained release profiles, and improved *in vivo* efficacy demonstrate that nano/micro-carriers can significantly amplify clove oil’s antibacterial performance. Continued development of such delivery systems is likely crucial to translate clove’s *in vitro* promise into clinical reality. Finally, a strategic shift in antibiotic discovery is imperative to mitigate the rapid emergence of resistance. Future efforts must transition from the modification of established antibiotic scaffolds and their known bacterial targets toward the identification of novel chemotypes with unprecedented mechanisms of action (Aminov, 2025). In this pursuit, exploiting the poly-pharmacological synergy inherent in medicinal plant molecules could provide a viable pathway to novel therapeutic strategies that circumvent pre-existing resistomes.

## 8 Limitations of the study

While this review brings together extensive *in vitro* and preliminary *in vivo* evidence on clove-derived phytochemicals, several limitations temper the strength of our conclusions. First, the preponderance of data arises from laboratory assays, such as broth microdilution and biofilm inhibition tests, that, although invaluable for elucidating mechanistic insights, cannot fully reproduce the complex interplay of host immunity, tissue distribution, and pathogen virulence encountered in living organisms. Consequently, the actual efficacy and safety profiles of cloves constituents in systemic infections remain uncertain until larger, well-designed animal experiments and human clinical trials are conducted.

The diversity of extraction and formulation techniques reported across studies, ranging from steam distillation and supercritical CO₂ extraction to various nano-emulsion and liposomal encapsulation methods, also introduces significant heterogeneity in phytochemical composition and bioavailability. Without standardized sourcing, processing, and reporting protocols, it is difficult to compare results directly or to establish reproducible dosing regimens for future investigations. In parallel, pharmacokinetic parameters for major clove compounds, especially eugenol, are inadequately characterized; critical information on tissue penetration, metabolic half-lives, and clearance pathways is largely absent from the literature, undermining the ability to predict therapeutic windows or potential toxicities.

Moreover, most synergy studies highlighting dramatic reductions in antibiotic minimum inhibitory concentrations (MICs) may be subject to publication bias: positive interactions are preferentially published, whereas neutral or antagonistic findings receive less attention. This skew could overstate the generalizability of phytochemical–antibiotic combinations. Additionally, antimicrobial testing methods vary widely, such as disk diffusion versus microdilution, differing inoculum sizes, and incubation conditions, which further complicates efforts to synthesize MIC values into clinically meaningful breakpoints. Finally, the current focus on a limited set of pathogens (*E. coli*, *S. aureus*, *K. pneumoniae*, *P. aeruginosa*) leaves critical gaps regarding emerging multidrug-resistant species and the potential impact of clove derivatives on the broader host microbiome or the evolution of resistance.

Addressing these limitations will require coordinated efforts to standardize extract preparation and assay protocols, to expand *in vivo* and clinical testing, and to integrate robust pharmacokinetic and toxicological studies. Only then can clove phytochemicals be credibly advanced as adjunctive therapies against MDR bacterial infections.

## 9 Conclusion

Cloves and its principal phytochemicals present in its essential oils and extracts, mainly eugenol and other minor components like eugenol Acetate, *β*-Caryophyllene, *α*-Humulene which could interact synergistically, exhibit broad-spectrum, multi-target antibacterial and anti-virulence activities against a wide array of MDR pathogens. Mechanistically, those clove-derived compounds disrupt bacterial membranes, inhibit efflux pumps, interfere with key metabolic pathways, and attenuate quorum-sensing–mediated virulence, positioning them as promising multi-target agents distinct from conventional antibiotics. Beyond direct antibacterial potency, the concept of “synergistic selectivity” emerges as a compelling clinical strategy: when combined with conventional antibiotics, clove phytochemicals can sensitize pathogens, allowing lower antibiotic doses to achieve equivalent or superior efficacy “disarm, then treat,” thereby expanding the therapeutic window and reducing host toxicity, an especially critical consideration for last-line drugs such as colistin.

Despite robust *in vitro* evidence, significant translational gaps remain. Key limitations include poorly characterized pharmacokinetics (rapid absorption but extensive metabolism and low systemic bioavailability for eugenol), variable extract standardization, heterogeneous assay methodologies, and limited *in vivo* and clinical data. Early animal studies and preliminary clinical observations (e.g., improved wound healing, reduced ventilator-associated pneumonia, and biofilm inhibition) are encouraging but insufficient to support routine clinical use. Formulation science is now recognized as a central bottleneck: rational delivery systems, including nanoemulsions, liposomes, and solid lipid nanoparticles, are required to achieve controlled local concentrations, sustained exposure, and acceptable safety profiles *in vivo*.

To advance clinical translation, we recommend prioritized efforts in three interrelated areas: (i) standardized, reproducible formulation and dose-finding studies integrating pharmacokinetics and pharmacodynamics (PK/PD); (ii) rigorous *in vivo* efficacy and safety studies that quantify therapeutic index improvements achieved via phytochemical–antibiotic combinations; and (iii) prospective clinical trials targeting high-impact, last-line scenarios where dose reduction could meaningfully mitigate toxicity. By addressing these gaps, future research can bridge the divide between promising bench science and safe, effective clinical applications, establishing clove-derived bioactives as viable adjuncts (or even alternatives) in the management of multidrug-resistant bacterial infections.
